# Microbial Characterization of Yellow Curing Process of Codfish

**DOI:** 10.1155/2021/6072731

**Published:** 2021-11-03

**Authors:** Susana Dias, Lélia Chambel, Rogério Tenreiro, Leonor Nunes, Virgílio Loureiro

**Affiliations:** ^1^Polytechnic of Coimbra, Coimbra Agriculture School, Department of Agricultural Sciences and Technologies, Bencanta 3045-601, Coimbra, Portugal; ^2^Research Center for Natural Resources, Environment and Society (CERNAS), Coimbra Agriculture School, Bencanta, 3045-601 Coimbra, Portugal; ^3^BioISI—Biosystems and Integrative Sciences Institute, Faculdade de Ciências da Universidade de Lisboa, Lisbon, Portugal; ^4^CIIMAR/CIMAR, Interdisciplinary Center of Marine and Environmental Research, University of Porto, Terminal de Cruzeiros de Leixões, Av. General Norton de Matos, S/N4450-208 Matosinhos, Portugal; ^5^Instituto Superior de Agronomia, Lisbon, Portugal

## Abstract

Yellow cured codfish has a typical yellow colour, distinctive taste, and low salt content due to its special curing process of the raw salted codfish involving several soaks in water of the raw salted codfish, alternated with drying steps. The purpose of this study was to assess the main functional groups of bacteria involved in this process and relate them with physicochemical properties of the product. A total of 28 codfish from Iceland were supplied by two local companies. Seven stages of the curing process were analyzed. From each of these seven stages, four fish samples were collected to carry out the microbial and physicochemical analyses (moisture, salt content, pH, total volatile basic nitrogen (TVB-N), and trimethylamine nitrogen (TMA-N)). Bacteria counts were performed using the MPN method and adequate culture media for aerobic, proteolytic, sulphite-reducing, biogenic amine, and trimethylamine-producing and ammonifying bacteria. Strains isolated from the highest dilutions with microbial growth were used to characterize the predominant bacteria. The results showed that total aerobic counts increased from 3.9 log MPN/g in raw salted codfish to 5.9 log MPN/g in the final. Proteolytic, ammonifying, and trimethylamine bacteria producers also increased to 8, 7.5, and 6.5 log MPN/g, respectively. The salt content decreases (from 17% until 8%) and moisture increases (53% until 67%) during the salted-raw-codfish soaking, favoring sulphite-reducing and biogenic amine-producing species, confirming that desalting enhances potential spoilers. The subsequent drying step benefits proteolytic, ammonifying, and trimethylamine-producing bacteria, with a corresponding non-protein-nitrogen content (TVB-N and TMA-N) increase. The dominant bacteria during yellow curing belong to the genera *Staphylococcus*, *Psychrobacter*, *Pseudomonas*, and *Alcaligenes* with a clear positive correlation between the content of *Staphylococcus* and *Psychrobacter* and TVB-N and TMA-N concentration. *Staphylococcus* spp. are the dominant bacteria in the steps where the product has a higher salt concentration; thus, it could be particularly useful as an indicator to control the industrially yellow curing process and could have an important role in the development of the final characteristics of this product.

## 1. Introduction

The salt cod preservation was introduced by the Basque people, based on the methodology applied to whale meat [1]. Salted cod has been the basis of several culinary recipes in Mediterranean gastronomy. Portuguese yellow cured cod fish stands upon the Newfoundland methodology of light curing, and this knowledge was brought to Europe by the Portuguese fishermen [2]. This kind of methodology confers an amber colour, a lower salt content, and a typical flavour to the final product. Presently, according to Portuguese legislation (DL no. 25/2005 28 of January), yellow cure codfish is a product with 12-16% salt content and 45% moisture after drying with a characteristic yellow colour.

In the traditional method, the raw salted fish is washed and submitted to soaking, which can be for several hours, and successive drying and press-pilling periods [3]. As this cure is performed with a lower amount of salt, the fish becomes more predisposed to microbial development, mainly slime-forming bacteria. Thus, it is imperative to dry this raw material immediately after soaking, usually at about 27°C in an atmosphere having about 50-55% of relative humidity in the first drying period and 60-65% over the subsequent ones. The drying periods must be carried out for 12 h [3, 4].

The slime formation in the yellow cure could be observed through the press-pilling period and also in the initial stages of drying. The flesh surface of the fish presents a layer of semigreasy and sticky glistening substance, and the odor is very characteristically strong and pungent [5].

A study performed by Dussault [5] about the bacteriology of this yellow curing reveals that during the first few days of curing, there was a sharp increase of total microorganisms, from 10^4^ to 10^5^ per cm^2^, of the flesh surface, followed by a gradual decline during the subsequent drying operation. Predominant microbiota consisted of Micrococci, *Achromobacter*, *Flavobacterium*, *Pseudomonas*, and *Sarcina*. Many of the Micrococcus strains exhibited an increasing proteolytic activity up to a maximum in 2% NaCl and declining to zero in 12% NaCl, and about 50% of the cultures were capable to reduce trimethylamine oxide (TMAO) to trimethylamine (TMA). This observation may lead to the assumption that the characteristic flavour and aroma of the yellow cure must be due to the activities of such Micrococci [5]. High levels of TMA and total basic volatile compounds (TBV) would be indicators of the bacterial spoilage [6, 7]. Free amino acids and TMA values in yellow cured cod are higher than in dry-heavy-salted cod which may be correlated with the difference in bacterial counts, those of yellow cured cod being much higher than those of dry-heavy-salted cod [4].

Ancient and some more recent studies relating to salted codfish bacteria were performed by several authors [5, 8–20]; however, there is very little information about the evolution of microbial quantity and diversity and the role played by these microorganisms throughout the yellow cure process.

There is a growing demand for yellow cured cod specially during festive seasons, and it has higher economic value than the white salted cod (dry-heavy-salted), which is evident when browsing commercial or restaurant sites (http://www.napoleao.eu/cc/portuguese-gourmet;http://culinarybackstreets.com/cities-category/Lisbon/2020/bacalhau), because of its unique sensorial characteristics greatly appreciated in the gourmet market as reported by Gomes et al. [21].

However, because of its higher risk of deterioration during process and storage, it is produced in small quantities requiring experienced handiwork.

The purpose of this study was to typify the main microbial functional groups along the curing process and establish microbiological indicators that are borderline between the preserved product and the product already in the spoiling phase related with physicochemical properties, which could became useful for the industrial process control. The findings shall contribute to interfere in the technological process that will make the yellow cure a scientifically controllable process and not, as it still happens today, an artisanal process based on empiricism causing many commercial losses.

## 2. Materials and Methods

**Figure 1:**
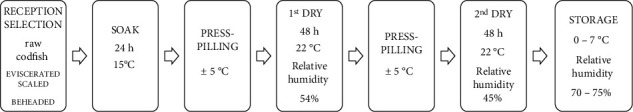
Diagram of the company's preparation of yellow curing cod.

A total of 28 codfish from Iceland were supplied by two local factories that produced them under commercial conditions illustrated in Figure 1.

Raw salted codfish already beheaded, opened, and gutted (without being dried) presented about 4 to 5 kg each. Selected specimens were immersed in water for 24 h, and after press-pilling, fish went to an artificial dryer for 48 h at 22°C under a relative humidity of 54% (1^st^ dry). Then, they were submitted to another pressing followed by another drying period at 22°C over 48 h under a relative humidity of 45% (2^nd^ dry). After this process, fish were stored at 0-7°C and under 75% of relative humidity (final product), until the first signs of deterioration (after 3 months) and the appearance of putrefaction with slime and unpleasant odors (after 5 months) were achieved. From each of these seven stages (raw codfish, after soaking, 1^st^ dry, 2^nd^ dry, final product, after 3 months, and after 5 months), 4 fish samples were removed to perform the microbial and physicochemical analyses.

Portions from the loin, tail, and wing muscle were aseptically taken from each fish, and 10 g of mixed samples was collected for a Stomacher bag containing 90 mL of tryptone saline solution (0.1% of tryptone—Scharlau 07-154 and 1% of NaCl). After homogenization in a Stomacher for 4 min, a series of tenfold dilutions were made.

Bacteria counts were performed using the most probable number (MPN) procedure and adequate culture media: aerobic mesophilic (yeast extract—Scharlau 07-079-2.5 g; tryptone 5 g; glucose 1 g—Scharlau; 1% NaCl), proteolytic and ammonifying bacteria using media described by Pochon and Tardieux [22], sulphite-reducing bacteria (Iron Media of Lyngby—Oxoid CM0964 without agar), histamine-forming bacteria using media developed by Niven et al. [23], and TMA-producing bacteria in growth media broth with oxitrimethylamine [24]. Counts were done after 4 days of incubation at 28°C, according to preliminary studies not published. For proteolytic and ammonifying bacteria, incubation time was 15 days [22]. All results were expressed as log MPN log/g of fish.

### 2.1. Phenotypic Characterization of Predominant Strains

Aliquots of 0.1 mL, from the highest dilutions with microbial growth in each liquid culture media, were spread in corresponding agar media to characterize and select the predominant bacteria colonies. Isolates were purified and submitted to a simple phenotypic characterization by Gram staining reaction, cellular morphology, and catalase and cytochrome oxidase activity. The 53 isolated strains were further tested for growth at different temperatures (15, 30, and 37°C) and different NaCl concentration (0, 1, 10, and 15%) in nutritive agar (Oxoid CM003).

### 2.2. Bacterial DNA Extraction from Isolated Strains

Biomass from purified bacteria cultures on nutritive agar (Oxoid CM003) was transferred to tubes containing 10 mL of 0.3% saline sterile solution to achieve a 5 McFarland Standard cell suspension; then, 1 mL was transferred to microcentrifuge tubes (Microfuge 18 Beckman) and centrifuged for 5 min at 10,000 rpm.

The recovered cells were washed with the saline solution. For cell lyses, the suspensions were frozen at -20°C and then heated at 100°C for 10 min. The suspension obtained was immediately centrifuged. After 5 min at 10,000 rpm, 1 *μ*L of lysed cell preparation was used in a PCR reaction with the Primer CsM13 [25]. The amplification was performed in a thermocycler (Biometra), according to Chambel et al. [26].

Visualization of PCR products was performed in a horizontal electrophoresis chamber (Pharmacia) with an Electrophoresis Power Supply EPS 600. DNA fragments were stained with 0.2 mg/mL of ethidium bromide and visualized with an ultraviolet transilluminator and Image system Alliance 4.7 Uvitec Cambridge. Data images were analyzed with the BioNumerics 6.6 software.

### 2.3. 16S rRNA Gene Sequencing of Isolated Strains, Computational Data, and Sequence Analysis

Based on isolate profiles and BioNumerics analysis, 31 strains from representative clusters were selected (data not shown) and used for amplification of the 16S rDNA with the primers PA 5′-AGAGTTTGATCCTGGCTCAG-3′ [27] and 1392R 3′-ACGGGCGGTGTGTRC-5′ [28] or 907R 3′-CCGTCAATTCMTTTRAGTTT-5′ [29].

From each selected strain, 1 *μ*L of DNA was mixed with 5 *μ*L of 10x buffer (60 mM (NH_4_)_2_SO_4_, 670 mM Tris HCl pH 8.8, 0.1% Tween 20), 2 *μ*L of 50 mM MgCl_2_, 1 *μ*L of 10 *μ*M dNTPs, 1 *μ*L of 50 pmoles/*μ*L Primer PA, 1 *μ*L of 50 pmoles/*μ*L Primer 1392R (or 907R), and 0.2 *μ*L of 5 U/*μ*L Taq DNA polymerase (with W-1, Invitrogen). The PCR mixture was completed with sterilized Milli-Q water to 50 *μ*L of final volume.

The PCR amplification was performed in a Thermocycler (Biometra), according to Chambel et al. [26], and the PCR product purification was accomplished with the Jetquick Spin Column Technique PCR Purification Kit (Genomed) and tested by performing electrophoresis of the samples on agarose gel at 1% for 1 h at 90 V and further revelation with ethidium bromide as described above. The selected strains were sequenced with two primers 104F (GGCGVAYGGGTGAGTAA) and 907R [30].

Sequences were assembled from the corresponding chromatograms using Chromas 2.33 and BioEdit and sequence alignment using Clustal X2 and GeneDoc gd322700 (consensus sequence editing) to resolve any ambiguities. The resulting 16S rRNA sequences were compared with DNA sequences of the GenBank databases using the BLAST program (Basic Local Alignment Search Tool). A minimum of 500 to 525 bp sequenced and <1% position ambiguities and the 99% sequence similarity limit required to consider two strains as belonging to the same species were used [31].

### 2.4. Physicochemical Analyses

Samples of loin, tail, and wing codfish muscle were cut and homogenized per fish. Determination of moisture, salt content, pH, total volatile basic nitrogen (TVB-N), and trimethylamine nitrogen (TMA-N) was carried out in duplicate in each fish sample. Moisture and salt content was performed according to AOAC [32]. A pH-meter Metrohm 744 was used to determine pH values; TVB-N and TMA-N were analyzed according to the method described in [33] and [34], respectively.

For the free amino acids, nitrogen content determination was adopted using an internal method of IPIPMA—titrimetric method—that used 25 g of crushed muscle to prepare the extract with trichloroacetic acid solution of 20% (Scharlau, Barcelona, Spain), which was neutralized with NaOH 15% in the presence of phenolphthalein and formaldehyde of 37%. Results of TVB-N, TMA-N, and free amino acids were expressed as mg of N/100 g of fish muscle.

### 2.5. Statistical Analyses

The “one-way ANOVA” analysis of variance of the STATISTICA 7 program (stat-sof, USA, 2004) was performed, after checking the normality and homogeneity of variance, using the Tukey test, to verify the significant differences. When the assumptions were not confirmed, the nonparametric Kruskal-Wallis test was used. The level of significance was defined as 0.05 (*p* ≤ 0.05).

## 3. Results

### 3.1. Microbiological Results

**Figure 2:**
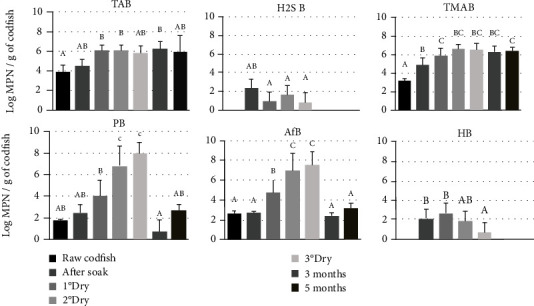
Change in abundance of bacterial different functional groups during the curing process: TAB: total aerobes; H2SB: sulphite-reducing bacteria/growth in iron agar; TMAB: trimethylamine-producing bacteria; PB: proteolytic bacteria; AfB: ammonifying bacteria; HB: histamine-producing bacteria. Different letters show statistically significant differences between processing stages (*p* < 0.05).

The results showed that total aerobic counts increased from 3.9 log MPN/g in raw salted codfish to 5.9 log MPN/g in the final product; proteolytic, ammonifying, and trimethylamine bacteria producers also increased to 8 and 7.5 and 6.5 log microorganisms/g, respectively (Figure 2). During salted codfish soaking, the sulphite-reducing and the biogenic-amine-producing bacteria are favored.

All groups of bacteria studied, defined as in Figure 2, were detected after the immersion step. However, after this stage, there were changes in the amount of microbial groups and some of which were no longer detected.

The product after the 2^nd^ drying and the final product were characterized by a high content of proteolytic, ammonifying, and TMA-producing microorganisms (Figure 2). The group of TMA producers reached the maximum values after the 2^nd^drying phase, remaining with minor changes until the end of 5 months of storage.

None of the strains isolated in the soaking stage showed growth with 15% salt (Table 1). These values increased with the beginning of deteriorated appearance that is detected after 3 months of storage, pronouncing up to 5 months, where 86% of the strains were Gram-positive and 71.4% developed in a medium with 15% NaCl.

All isolated strains showed the ability to grow at temperatures of 15°C and 30°C (Table 1).

At 37°C of temperature, it was evident that most of the isolates after first dry are able to grow.

**Figure 3:**
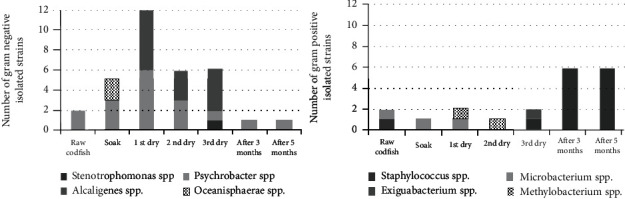
Distribution of the main isolated and identified Gram-negative and Gram-positive bacteria along the curing process and putrefaction of the yellow codfish.

The 16S rRNA Gene Sequencing of the isolated Gram-negative strains (Figure 3) revealed that the dominant genera were *Oceanisphaerae*, *Alcaligenes*, and *Psychrobacter*.

*Alcaligenes* spp. predominates in the initial drying and final product phases and was not detected when the putrefaction process started. *Psychrobacter* spp. can be found in all studied stages, including after 3 and 5 months of storage but with higher incidence in the first drying. Based on the sequenced Gram-positive strains (Figure 3), 60% were identified as *Staphylococcus* spp., and this genera is predominant in the stages in which the product has more salt, that is, in the raw and final curing and more evident in the stages after 3 and 5 months of storage.

### 3.2. Physicochemical Results

**Figure 4:**
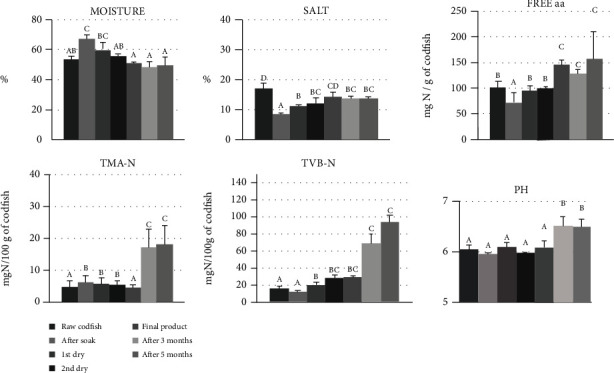
Changes in moisture, salt, TMA-N, free amino acids, TVB-N content, and pH along the curing process and putrefaction of the product. Different letters indicate that there are statistically significant differences between the stages (*p* < 0.05).

The moisture content reached the maximum value after soaking, decreasing significantly (*p* < 0.05) in the subsequent stages of drying and stayed below 50% after 3 months (Figure 4).

The salt content is significantly reduced by soaking, lowered to a value below 10%. Over the drying stages, there is a statistically significant increase (*p* < 0.05) but always presenting values lower than 15%, even after 3 and 5 months of storage (Figure 4).

During the yellow curing process, the pH remained around 6 until 3 months of storage, at which time, there was a statistically significant increase (*p* < 0.05) reaching 6.5. This pH increase was accompanied by a statistically significant increase (*p* < 0.05) of TBV-N, TMA-N, and free aminoacid content (Figure 4), achieving 90, 20, and 150 mg N/100 g of fish, respectively.

## 4. Discussion

In complex matrices as codfish, it is difficult to differentiate the main groups of viable microorganisms because their recovery is difficult and dependent on methods, culture media, and incubation conditions. Thus, it was decided to use the multiple tube method to understand the population dynamic of microbial groups. This analysis was focused on the tubes at higher dilutions with positive growth in order to establish the dominant bacterial genera in the different stages of the curing process. The results allowed the quantification of the main microbial groups throughout the process and identification of the most relevant microbial genera.

Concerning the main microbial group quantification, higher values of standard deviation were obtained (Figure 2) reflecting the complexity of the samples. Raw salted codfish had a microbial count of less than 10^5^ cells/g which is quite lower comparing to fresh fish [35, 36] and suggesting that the effect of the salt was effective in the inhibition or death of a large part of the microbiota present before salting.

Expectedly, moisture of codfish increases with soaking and the salt concentration decreases in this stage because it is removed by the water. There is a predominance of Gram-negative strains in the soaking stage which had already been recognized by Pedro et al. [37] and found in the work of Rodrigues et al. [14]. The Gram-negative strains were identified as belonging to *Alcaligenes* spp. and *Psychrobacter* spp. Although the strains were identified with accuracy only up to the level of the genus, it is possible to admit that these bacteria come from the marine environment, which constitute the cod habitat because most species of these genus are typical of cold environments both marine or terrestrial [6, 36, 38–40].

During the drying stage, the rise in non-protein-nitrogen is coincident with the significant increase in the percentage of salt due to the decrease in moisture. Dryer temperatures could act as a selective factor, which explains that after the first drying, there is a higher percentage of strains capable of growing at temperature of 37°C (Table 1) when compared to strains isolated in the previous stages. The genera *Alcaligenes* was predominant in the drying stage, but it was possible to find *Psychrobacter* spp. in all curing stages, including in raw codfish and in the spoiled product, which is understandable since it is a halotolerant bacteria [38]. In general, members of the genera *Psychrobacter* exhibited lipolytic activity and are capable of hydrolyzing some amino acids such as leucine [41]. According to Garcia-Lopez and Maradona [42], they are not associated with TMA production in large quantities, but there are at least two species, *P. cibarius* and *P. maritimus*, which showed slight TMA production during storage at 4°C. Although this TMA was produced slightly, the presence of *Psychrobacter* spp. may be related to the unpleasant odor released in spoiled fish [41]. During the second drying, an increase of one order of magnitude of proteolytic bacteria was observed (Figure 2) which was represented by *Psychrobacter* spp. (Figure 3), which seems to be the main protagonist of the curing process.

After 3 months of storage, the formation of the characteristic slime and the repulsive smell denounces an intense microbial activity with the formation of ammonia and trimethylamine proven by the statistically significant increase (*p* < 0.05) in the most relevant physicochemical parameters, such TVB-N and TMA-N, and pH, in codfish after 3 and 5 months of storage. The increase of pH is in accordance with what was already described by Botelho [43] who also found that the slime formation was followed by the rise in pH.

The evaluation after 3 months of storage showed that there was a significant decrease (*p* < 0.05) in the content of groups of proteolytic and ammonifying bacteria, which remained over the state of putrefaction. In fact, in the cod samples after 3 and 5 months, muscle decomposition was not observed, contrary to what would be expected given the slime formation and unpleasant and pungent smell. This makes evident that proteolysis has not reached an extreme degree that would cause the tissue disintegration. Another possible justification for this decrease in microorganism content is the difficulty in the extraction of microorganisms from the polysaccharide matrix which could decrease its quantification. In the putrefaction phase, the bacterial groups that prevailed were those that use the compounds resulting from proteolysis, biosynthesizing other nitrogen compounds responsible for the changes of the sensorial characteristics [44–47], namely, the TMA producers and ammonifying bacteria despite a significant (*p* < 0.05) reduction of the content of the latter group.

Regarding the whole process, the major percentage of Gram-positive isolated strains was identified as *Staphylococcus* spp., and they were found not only in the raw codfish but also in codfish after 3 and 5 months; they were predominant. However, it was not found in the soaking stage. All isolated strains grew at 10% salt and 37°C, thus revealing the ability to resist the high salt concentrations of the product and the drying temperatures used in the process. Some strains of this genus were already used as starter cultures in curing meat products, known for their proteolytic activity, which results in high non-protein-nitrogen contents [48].

Concerning products with at least 3 months, a higher prevalence of the genera *Staphylococcus* coagulase negative was detected. Of the strains identified as belonging to the genera *Staphylococcus*, no species were identified as *Staphylococcus aureus*. The isolated strains were identified as belonging to the species *Staphylococcus epidermis* (homology between 94 and 98%) or to the species *Staphylococcus equorum* (homology 94 to 99%).

Although *Staphylococcus* spp. was the dominant genera, to justify its use as a yellow cured codfish spoilage microbiological indicator, the involvement of other genera of bacteria including other Gram-positive bacteria must be admitted.

## 5. Conclusion

The yellow curing of cod involves high microbial activity evident by the changes in the functional group levels and physicochemical parameters especially pH, and TVB-N and TMA-N contents. The most important functional groups involved were as follows: proteolytic bacteria, namely, *Psychrobacter* spp. (which is active throughout the process); TMA-producing bacteria, such as *Psychrobacter* spp., *Alcaligenes* spp., and *Staphylococcus* spp.; and ammonifying bacteria.

*Staphylococcus* spp. was found to be the dominant bacteria in the processing stages where the product has a higher salt concentration. Based on our results, the genera *Staphylococcus* detection and quantification may be particularly useful as a microbial indicator to control the industrially yellow curing process and could have an important role in the development of the final characteristics of this product.

It was in the middle of the 20th century that there was a greater interest in learning more about the yellow curing process. The commercial status of yellow cured cod and the current added value that it represents as a *gourmet* delicacy justifies further scientific studies. The present study was developed in this context allowing the acquired knowledge to be transferred to the industry so that production is more controlled while reducing losses.

## Figures and Tables

**Table 1:** Phenotypic characterization of microbial strains isolated according to the processing stage.

Processing stage	% Gram+	% catalase+	% oxidase+	15°C	30°C	37°C	% with growth at			
							NaCl 0%	NaCl 1%	NaCl 10%	NaCl 15%
Raw codfish (*n* = 4)	25	100	50	100	100	0	100	100	100	50
Soak (*n* = 6)	17	100	66	100	100	17	100	100	100	0
1^st^ dry (*n* = 14)	14	100	86	100	100	57	100	100	57	21
2^nd^ dry (*n* = 7)	14	100	71	100	100	71	100	86	29	14
3^rd^ dry or final product (*n* = 8)	25	100	75	100	100	100	100	100	50	13
After 3 months (*n* = 7)	86	100	14	86	86	86	86	86	86	71
After 5 months (*n* = 7)	86	100	0	100	100	100	100	100	100	71

*n*: number of strains isolated from each stage or each culture media.

## Data Availability

The corresponding author (sudias@esac.pt) will provide all the information about microbial and chemical data used to support the findings of this study upon request.

## References

[1] Kurlansky M., Pinheiro J. M. (2000). *O bacalhau, biografia do peixe que mudou o mundo*.

[2] Castro O. (1958). *Experiências de Cura Amarela do Bacalhau e seu possível interesse na secagem de peixe para climas quentes*.

[3] Legendre R. V. (1955). The artificial drying of lightly salted codfish. *Journal of the Fisheries Research Board of Canada*.

[4] Klaveren F. W., Legendre R., Borgstrom G. (1965). Salted cod. In: fish as food. *Processing-Parte 1*.

[5] Dussault H. P. (1953). Bacteriology of light salted fish-sliming. *J Fish Res Board Can. Progr. Repts. Atlantic Coast Stas.*.

[6] Shewan J. M., Borgstrom G. (1961). The microbiology of sea-water fish. *Fish as Food: Production, Biochemistry and Microbiology (Vol. I)*.

[7] Veciana-Nogués M., Mariné-Font A., Vidal-Carou M. (1997). Biogenic amines as hygienic quality Indicators of tuna. Relationships with microbial counts, ATP-related compounds, volatile amines, and organoleptic changes. *Journal of Agricultural and Food Chemistry*.

[8] Aas G. H., Skjerdal O. T., Stoknes I., Bjørkevoll I. (2010). Effects of packaging method on salt-cured cod yield and quality during storage. *Journal of Aquatic Food Product Technology*.

[9] Dyer F. (1947). Microorganisms from Atlantic cod. *Journal of the Fisheries Research Board of Canada*.

[10] Fernández-Segovia I., Escriche I., Fuentes A., Serra J. A. (2007). Microbial and sensory changes during refrigerated storage of desalted cod (Gadus morhua) preserved by combined methods. *International Journal of Food Microbiology*.

[11] Kuuliala L., Al Hage Y., Ioannidis A. G. (2018). Microbiological, chemical and sensory spoilage analysis of raw Atlantic cod (Gadus morhua) stored under modified atmospheres. *Food Microbiology*.

[12] Lorentzen G., Olsen R., Bjørkevoll I., Mikkelsen H., Skjerdal O. (2010). Survival of Listeria innocua and Listeria monocytogenes in muscle of cod (Gadus morhua L.) during salt-curing and growth during chilled storage of rehydrated product. *Food Control*.

[13] Pedro S., Albuquerque M., Nunes M. L., Bernardo F. (2004). Pathogenic bacteria and indicators in salted cod (Gadus morhua) and desalted products at low and high temperatures. *Journal of Aquatic Food Product Technology*.

[14] Rodrigues M. J., López-Caballero H. P., Vaz-Pires M., Nunes M. (2003). Characterization and identification of microflora from soaked cod and respective salted raw materials. *Food Microbiology*.

[15] Reynisson E., Lauzon H. L., Magnússon H. (2009). Bacterial composition and succession during storage of North-Atlantic cod (Gadus morhua) at superchilled temperatures. *BMC Microbiology*.

[16] Rodrigues M., Ho P., López-Caballero M., Bandarra N., Nunes M. (2006). Chemical, microbiological, and sensory quality of cod products salted in different brines. *Journal of Food Science*.

[17] Skjerdal O., Lorentzen G., e Joensen S. A. (1997). *Microflora in desalted cod*.

[18] Soares M., Neves A. (2017). Avaliação microbiológica de bacalhau salgado seco desfiado refrigerado no comércio retalhista. *Revista da Unidade de Investigação do Instituto Politécnico de Santarém*.

[19] Vilhelmsson O., Hafsteinsson H., Kristjánsson J. (1997). Extremely halotolerant bacteria characteristic of fully cured and dried cod. *International Journal of Food Microbiology*.

[20] Dussault H. P. (1962). Enumeration of coliform bacteria in light salted fish brines. *Journal of the Fisheries Research Board of Canada*.

[21] Gomes G., Sampaio J., Silva M., Reis M., FRANQUEIRA T. (2011). (Re)designing a traditional product-design strategies for the development of yellow codfish products and its positioning in gourmet markets. http://hdl.handle.net/10773/4371.

[22] Pochon J., Tardieux P. (1962). Techniques d’analyse em microbiologie du sol. *Collection Technique de Base*.

[23] Niven C., Jeffrey M., Corlett D. A. (1981). Differential planting medium for quantitative detection of histamine-producing bacteria. *Applied and Environmental Microbiology*.

[24] Dalgaard P., Ross T., Kampermann L., Neumeye K., McMeekin T. (1994). Estimation of bacterial growth rates from turbidimetric and viable count data. *International Journal of Food Microbiology*.

[25] Huey B., Hall J. (1989). Hypervariable DNA. Fingerprinting in Escherichia coli: minisatellite probe from bacteriophage M13. *Journal of Bacteriology*.

[26] Chambel L., Sol M., Fernandes I. (2007). Occurrence and persistence of Listeria spp. in the environment of ewe and cow's milk cheese dairies in Portugal unveiled by an integrated analysis of identification, typing and spatial-temporal mapping along production cycle. *International Journal of Food Microbiology*.

[27] Massol-Deya A. A., Odelson D. A., Hickey R. F., Tiedje J. M., Adl A., Van-Elsas J. D., de-Bruijn (1995). Bacterial community fingerprinting of amplified 16S-23S ribosomal gene sequences and restriction endonuclease analysis (ARDRA). *Molecular Microbial Ecol Methods*.

[28] Muyzer G., Brinkhoff T., Nubel U., Santegoeds C., Schaver H., ADL A., Van-Elsas J. D., FJ D.-B. (1998). Denaturing gradient gel electrophoresis (DGGE) in microbial ecology. *molecular microbial ecology methods*.

[29] Muyzer G., Hottentrager S., Teske A., Wawer C., ADL A., van-Elsas J. D., FJ D.-B. (1996). Denaturing gradient gel eletrophoresis of PCR-amplified 16SrDNA - a new molecular approach to analyse the genetic diversity of mixed microbial communities. *Molecular Microbial Ecology Methods*.

[30] Chaves S. (2005). *Diversidade de procariotas sulfato-redutores e desnitrificantes em amostras ambientais*.

[31] Janda M. J., Abbott S. L. (2007). 16S rRNA gene sequencing for bacterial identification in the diagnostic laboratory: pluses, perils, and pitfalls. *ASM journals on CD*.

[32] AOAC (2005). *Official Methods of Analysis*.

[33] IPQ (2009). *NP 2930 Fishery and Aquaculture Products; Determination of Total Volatile Basic Nitrogen*.

[34] IPQ (2009). *NP 1841 Fishery and Aquaculture Products; Determination of Trimethylamine Nitrogen (N-TMA) Content*.

[35] Austin B. (2006). The bacterial microflora of fish, revised. *Scientific World Journal*.

[36] Huss H. H. (1995). *Quality and quality changes in fresh fish*.

[37] Pedro S., Magalhães N., Albuquerque M., Batista I., Nunes L., Bernardo F. (2002). Preliminary observations on spoilage potential of flora from desalted cod (Gadus morhua). *Journal of Aquatic Food Product Technology*.

[38] Bozal N., Montes M. J., Tudela E., Guinea J. (2003). Characterization of several Psychrobacter strains isolated from Antartic environments and description of Psychrobacter luti sp. nov. and Psychrobacter fozzi sp. nov. *International Journal of Systematic and Evolutionary Microbiology*.

[39] Carneiro A. R., Ramos R. T., Dall’Agnol H. (2012). Genome sequence of Exiguobacterium antarcticum B7, isolated from a biofilm in Ginger Lake, King George Island, Antarctica. *Journal of Bacteriology*.

[40] Gram L., Huss H. H. (1996). Microbiological spoilage of fish and fish products. *International Journal of Food Microbiology*.

[41] Mejlholm O., Boknaes N., Dalgaard P. (2005). Shelf life and safety aspects of chilled cooked and peeled shrimps (Pandalus borealis) in modified atmosphere packaging. *Journal of Applied Microbiology*.

[42] Garcia-Lopez M., Maradona L. M., Robinson R. K., Batt C. A., Patel P. D. (2000). Psychrobacter. *Encyclopedia of Food Microbiology*.

[43] Botelho T., Herdeiro J. (1965). *Pescado seco e salgado*.

[44] Anagnostopoulos D. A., Kamilari E., Tsaltas D. (2020). Evolution of bacterial communities, physicochemical changes and sensorial attributes of natural whole and cracked Picual table olives during spontaneous and inoculated fermentation. *Frontiers in Microbiology*.

[45] Fadda S., López C., Vignolo G. (2010). Role of lactic acid bacteria during meat conditioning and fermentation: peptides generated as sensorial and hygienic biomarkers. *Meat Science*.

[46] Pinto F., Ponsano E., Franco M., Shimokomaki M. (2002). Charqui meats as fermented meat products: role of bacteria for some sensorial properties development. *Meat Science*.

[47] Zhao X., Zheng Z., Zhang J., Sarwar A., Aziz T., Yang Z. (2019). Change of proteolysis and sensory profile during ripening of cheddar-style cheese as influenced by a microbial rennet from rice wine. *Food Science & Nutrition*.

[48] Bover-Cid S., Izquierdo-Pulido M., Vidal-Carou C. (2001). Effect of the interaction between a low tyramine-producing Lactobacillus and proteolytic staphylococci on biogenic amine production during ripening and storage of dry sausages. *International Journal of Food Microbiology*.

